# Reproducing FSL's fMRI data analysis *via* Nipype: Relevance, challenges, and solutions

**DOI:** 10.3389/fnimg.2022.953215

**Published:** 2022-07-26

**Authors:** Yibei Chen, Frederic R. Hopp, Musa Malik, Paula T. Wang, Kylie Woodman, Sungbin Youk, René Weber

**Affiliations:** ^1^Media Neuroscience Lab, Department of Communication, College of Letters and Science, University of California, Santa Barbara, Santa Barbara, CA, United States; ^2^Amsterdam School of Communication Research, University of Amsterdam, Amsterdam, Netherlands; ^3^Department of Communication and Media, Ewha Womans University, Seoul, South Korea

**Keywords:** Nipype, FSL, fMRI analysis, reproducibility, methods

## Abstract

The “replication crisis” in neuroscientific research has led to calls for improving reproducibility. In traditional neuroscience analyses, irreproducibility may occur as a result of issues across various stages of the methodological process. For example, different operating systems, different software packages, and even different versions of the same package can lead to variable results. Nipype, an open-source Python project, integrates different neuroimaging software packages uniformly to improve the reproducibility of neuroimaging analyses. Nipype has the advantage over traditional software packages (e.g., FSL, ANFI, SPM, etc.) by (1) providing comprehensive software development frameworks and usage information, (2) improving computational efficiency, (3) facilitating reproducibility through sufficient details, and (4) easing the steep learning curve. Despite the rich tutorials it has provided, the Nipype community lacks a standard three-level GLM tutorial for FSL. Using the classical Flanker task dataset, we first precisely reproduce a three-level GLM analysis with FSL *via* Nipype. Next, we point out some undocumented discrepancies between Nipype and FSL functions that led to substantial differences in results. Finally, we provide revised Nipype code in re-executable notebooks that assure result invariability between FSL and Nipype. Our analyses, notebooks, and operating software specifications (e.g., docker build files) are available on the Open Science Framework platform.

## Introduction

The challenge to reproduce research findings has been a long-standing issue in many scientific fields, including neuroscience (Baker, 2016). In order to improve the reproducibility of neuroimaging results, recent calls emphasize stronger commitments toward open science by improving the transparency of design decisions, data, methods, and analytic approaches (Poldrack and Poline, 2015; Gorgolewski and Poldrack, 2016; Gilmore et al., 2017; Gleeson et al., 2017; Gorgolewski et al., 2017; Poldrack, 2019; Wagner et al., 2022). Focusing on the reproducibility of data analytic pipelines, previous research (Tustison et al., 2014; Glatard et al., 2015; Dickie et al., 2017; Kennedy et al., 2019; Botvinik-Nezer et al., 2020) has identified that even slight variations in analytic decisions (from the operating system and software choices to statistical modeling) can diminish reproducibility. For example, *libmath*—mathematical operations manipulating single-precision floating-point numbers—has evolved across different versions of GNU/Linux-based operating systems, leading to numerical differences in computational results. While those differences are often negligible at individual steps, their accumulating effects frequently lead to substantially different results in commonly-used analyses, including independent component analysis (ICA) (Glatard et al., 2015). Similarly, different neuroimaging software, such as FreeSurfer (Fischl, 2012) and Advanced Normalization Tools (ANTs, Avants et al., 2009), produces variable predictions of age and gender based on cortical thickness. This poses problems when age and gender are important imaging biomarkers of cognition, phenotype, or disease (Tustison et al., 2014). In addition, different statistical methods chosen by different research teams to test the same hypotheses on the same dataset can lead to variable conclusions (Botvinik-Nezer et al., 2020).

In view of these issues, being as transparent and detailed as possible about operating system, software, and analytic steps is instrumental for reproducibility (Gorgolewski and Poldrack, 2016). Therefore, this paper aims to contribute to the transparency and reproducibility of brain imaging data analysis in the following ways: First, we develop, compare, and offer re-executable Python-based Jupyter (https://jupyter.org/) notebooks for a three-level general linear model (GLM) based on Nipype (http://nipy.org) and FSL (https://fsl.fmrib.ox.ac.uk/). Although there are “blog-style” online tutorials and scripts^1^, ^2^, ^3^ for performing individual steps in univariate brain imaging, there currently exists no thoroughly documented, quality-controlled and peer-reviewed, easily re-executable workflow for a three-level GLM in FSL *via* Nipype. Second, we perform our analyses in an openly available computing environment to minimize variability in results caused by differences in operating system and software versions. Third, by using an openly available dataset, our analyses can directly be reproduced and lend themselves for instructional purposes in neuroimaging courses.

## Challenges using Nipype and FSL

Computational methods are nowadays an essential part of data analysis in all sciences; therefore, programming, software development, and computational thinking are becoming increasingly important in scientific practice (Wilson, 2006). Nipype (Gorgolewski et al., 2011), a neuroimaging library in Python, has witnessed a rapid increase in the number of studies using it. This popularity can at least partially be attributed to Nipype's uniform interface to existing neuroimaging software, such as ANTs, FSL, and SPM. In typical neuroscience practice, researchers tend to incorporate different software packages at different stages of the analysis. Examples include using ANTs for registering structural MRI images into the standard MNI space, or FSL and SPM for statistical analysis. Those packages, however, are accessed and integrated through multiple ways, such as shell scripting (FSL, ANTs), the MATLAB compiler for SPM, or dedicated graphical user interfaces (GUI, e.g., FSL). A common consequence of this variable integration of analytic tools is the difficulty to reproduce the exact analytic sequence using the exact same configurations. To counter this problem, Nipype presents a uniform and standardized interface in which analysts can use functions from diverse neuroimaging tools under the framework of a unified programming language—Python.

Introductions to Nipype's architecture are made available *via* a few hands-on tutorials developed by the neuroimaging community, covering its basic components (e.g., interface, node, and mapnode) and exemplary workflows (e.g., preprocessing and first-level analysis), to advanced usage (e.g., cloud computing). Notwithstanding their instructional value, these tutorials remain limited in important ways: First, tutorials frequently do not feature quality-controls that assess whether a particular result produced by Nipype replicates results produced by a typical workflow using stand-alone software. Because tutorials are typically reduced to “minimal working examples”, many additional—and often implicit—steps executed when running analyses with a full-stack GUI software library (e.g., SPM, FSL) are discarded. Hence, variable results are likely between simplified Nipype tutorials vs. comprehensive neuroimaging suites. Considering that a majority of neuroscientists still rely on GUI-guided analysis pipelines, it is vital that Nipype tutorials accurately mirror these analyses. Second, tutorials are frequently geared toward learning individual steps and components, but yet need to cover more comprehensive analyses that can accommodate common paradigms spanning multiple subjects and runs. Among the oldest and most frequently used statistical models for the analysis of neuroimaging data are multi-level GLMs (Friston et al., 1995; Worsley and Friston, 1995). Yet, a comprehensive tutorial covering a standard three-level GLM in Nipype is not available. To fill this gap, we introduce a series of tutorials using the FSL suite *via* Nipype.

Focusing on fMRI data analyses, FSL has implemented GLMs for fMRI data analysis in a powerful tool—FEAT (FMRI Expert Analysis Tool). FEAT includes advanced three-level GLM and has become a popular choice for neuroimaging analysts (Jenkinson et al., 2012). Integrating FSL/FEAT analysis into a flexible but standardized analytical pipeline *via* Nipype is desirable due to the reasons mentioned above. However, if we decide to use Nipype (and as some fellow analysts have done in the recent past; e.g., Esteban et al., 2020), we have to assume that running FSL/FEAT separately *via* scripting or its GUI and running it *via* Nipype lead to the exact same results. Surprisingly, this was not the case using the available online tutorials and documentation, despite using a standardized computing environment.

Therefore, the work presented here contributes to the neuroimaging community by precisely reproducing the results of a three-level GLM analysis with FSL/FEAT *via* Nipype, using the classical Flanker task dataset (Kelly et al., 2008). After standardizing the operating system and the software versions, we reveal undocumented discrepancies between the Nipype-based and the standalone FSL/FEAT functions that can lead to substantial differences in final results. We then provide revised Nipype code in re-executable Jupyter notebooks that assure result invariability between Nipype-based and standalone FSL/FEAT analyses. Our analyses, result comparisons, notebooks, and operating software specifications (e.g., Docker file) are available on the Open Science Framework platform^4^.

## Materials and methods

### Procedure

We used the Flanker dataset (described below) from OpenNeuro^5^ for our demonstration. We analyzed the dataset *via* a classic three-level GLM. For each GLM level, we first conducted the analysis with the FSL GUI, following a popular, step-by-step online tutorial^6^. Notably, FSL generates a detailed log file of all executed operations. We used this log file as a template, manually converting each command into Python to interface with Nipype's FSL packages. Finally, we compared all generated outputs between FSL GUI and Nipype.

### Dataset description

The Flanker dataset (Kelly et al., 2008) comprises data from 26 healthy adults who performed a slow event-related Eriksen Flanker task in two 5-min blocks (i.e., runs). Each run contains 12 congruent and 12 incongruent trials, presented in a pseudorandom order. On each trial of the task, participants used one of two buttons on a response pad to indicate the direction of a central arrow in an array of 5 arrows. The central arrow is either pointing in the same direction (congruent) or opposite direction (incongruent) of the 5-arrow array.

Functional imaging data were acquired using a Siemens Allegra 3.0 T scanner, with a standard Siemens head coil, located at the New York University Center for Brain Imaging. This dataset contains 146 contiguous echo planar imaging (EPI) whole-brain functional volumes (TR = 2,000 ms; acquisition voxel size = 3 × 3 × 4 mm) for each of the two runs. A high-resolution T1-weighted anatomical image was acquired using a magnetization prepared gradient echo sequence (TR = 2,500 ms).

### Computing environment

The three-level GLM was carried out *via* the FSL GUI (version 6.0.4) on a Linux workstation (Ubuntu 20.04.4 LTS). The FSL Nipype equivalent of the three-level GLM was run within a Docker container (version 20.10.12), generated *via* Neurodocker^7^. Neurodocker generates all specified (neuroimaging) software—including version specifications—in a stand-alone container, thereby allowing researchers to export their exact computing environment. Importantly, we ensured that the Docker container and our Linux desktop workstation had the same operating system and FSL version. Additional Python-based computing libraries included in the Docker container can be found in the corresponding docker file^8^.

### FMRIB software library

FSL can be accessed through both the GUI and the shell script. In this study, we used the FSL GUI to generate results from the three-level GLMs, supplemented by its log file that we transformed into an FSL shell script to generate files for step-wise comparisons with the Nipype outputs, as otherwise the FSL GUI would automatically overwrite files at each step.

FSL provides comprehensive analysis tools (e.g., BET and FEAT) for functional, structural and diffusion MRI brain imaging data (Smith et al., 2004). Here, we focused on its tools for functional MRI, specifically, FEAT–a complete tool for model-based fMRI analysis. FEAT carries out data preprocessing, first-level GLM analysis (i.e., within-run level); registration to subject-specific structural images and standard space; and higher-level GLM analyses (i.e., within-subject-cross-run and cross-subject level) on task-based functional images. In our case, there were two runs in our dataset, so each subject had two sets of functional images. We also used the Brain Extraction Tool (BET) on each subject's structural image before FEAT to generate subject-specific structural images.

### General setup of Nipype

Nipype has three major components (Gorgolewski et al., 2011): (1) *interfaces* to external tools (e.g., FSL) for setting up inputs, executing, and retrieving outputs; (2) a *workflow engine* to connect inputs and outputs of interfaces as a directed acyclic graph (DAG); and (3) *plug-ins* to execute workflows locally or in a distributed system. In the present project, we advance the usage of the *workflow* that connects inputs and outputs of FSL (*interface*) for the three-level GLMs in a local environment (*plug-in*).

To use FSL functions in Nipype, we wrapped those functions in either Node or MapNode objects. In graph theory, a node is a fundamental unit of which graphs are formed; similarly, a Node or MapNode object is the basic unit of a Nipype workflow. We can view each Node or MapNode as a function of FSL; the difference between a Node and a MapNode is that a Node operates on a single input while a MapNode enables operations on multiple inputs. For example, a Node is suitable for processing the brain imaging data from a single run of a single subject, while a MapNode is a better choice for processing data from multiple runs of multiple subjects. A workflow can thus be visualized as a directed acyclic graph of connected Node and MapNode objects, in which the connective sequence of the graph reflects the necessary chronological order of each functional step. Further details regarding setting up an analytical pipeline in Nipype can be found at https://nipype.readthedocs.io.

### The first-level GLM

Conducting a first-level (within-run) GLM analysis includes two steps: data preprocessing and within-run GLM time series analysis. The inputs are participants' structural and functional images plus the event information that indicates the onset, duration, and condition type of each trial.

In FSL, we first conducted a series of analyses on two runs of a single participant, and obtained the setup file “*design.fsf”* for each run. With these two setup files as the template, we used scripting and performed the same analysis for two runs of all 26 participants^9^. Specifically, we performed brain extraction *via* BET on the structural brain images with a fractional intensity threshold of 0.5. Then, using the functional images, the full first-level analysis *via* FEAT included data preprocessing and statistical modeling with a GLM. The preprocessing involved: motion correction (MCFLIRT), spatial smoothing (full width half maximum, FWHM, as 5 mm), temporal filtering (highpass cutoff 100 s), registration to the structural image (output from BET) and standard space (MNI152 T1-weighted 2 mm brain)^10^ For the statistical modeling–using the “Stats” tab in the FSL GUI–we set up four t-contrasts: (1) incongruent > baseline, which is the average beta weight for the incongruent condition compared to the baseline; (2) congruent > baseline, the average beta weight for the congruent condition compared to the baseline; (3) incongruent > congruent, which is the difference of the average beta weights between the incongruent and congruent conditions, highlighting the activation from the incongruent condition; and (4) congruent > incongruent, which is similar to (3) but highlights the activation from the congruent condition. We kept all other settings at their default.

**Figure 1:**
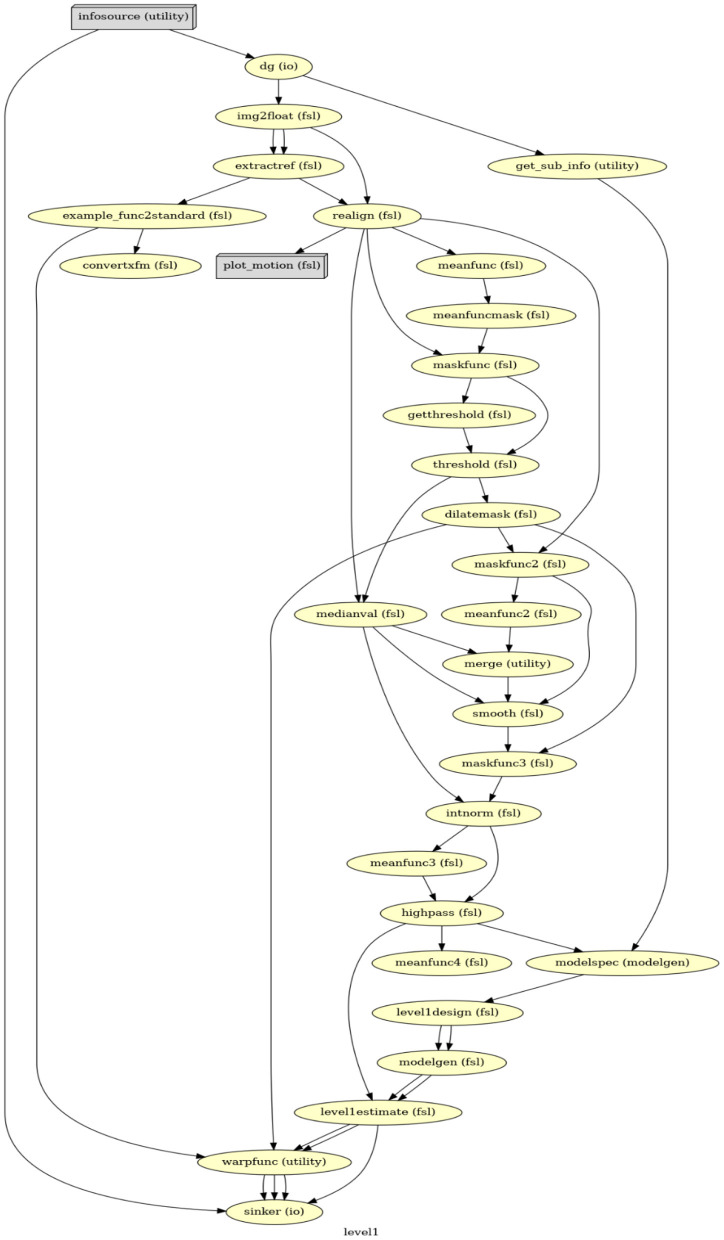
The directed acyclic graph (DAG) for the first-level GLM in Nipype. Everything before *modelspec* is about data preprocessing. From *modelspec* to *level1estimate* are the first-level model setup. The *warpfunc* represents transforming the first-level outputs to the standard space.

FSL saves results in a FEAT folder^11^ along with a log file, providing detailed shell scripts commands of settings in the GUI. This log file was then used as a guide to create the first-level GLM in Nipype. Our Nipype first-level GLM also includes data preprocessing and statistical modeling but in a more efficient and flexible manner. Firstly, instead of analyzing a single participant's data to generate the template for all participants, Nipype can simultaneously process data from all participants *via* a MapNode. Secondly, the FSL GUI requires performing BET before FEAT, but only uses the BET result at the late stage of FEAT; in contrast, Nipype can organize all functions (i.e., MapNodes/Nodes) in a chronological order based on their operative roles (Figure 1). Lastly, with Nipype the output structure can be customized, thereby effectively saving storage space. Whereas, FSL saves all files that were generated, in Nipype we can choose to only retain those that are related to parameter estimations and are necessary for a second-level GLM. Important output files that serve as input in the second-level analysis include the contrast of parameter estimates (cope) for each contrast, the variance of parameter estimates (varcope) for each contrast, and the binary brain mask. Therefore, we have four copes, four varcopes, and one mask per run for each participant.

### The second-level GLM

The second-level (within-subject-cross-run) GLM generally have two major parts: (1) warping the results (i.e., statistical images, such as copes, and varcopes) of the first-level GLM onto a standard template, and (2) setting up and fitting a fixed-effects model, which averages parameter estimations across two runs within each participant. Accordingly, the inputs of a second-level GLM are the results from the first-level GLMs.

In the FSL GUI, warping parameters are set up in the first-level GLM menus under “Registration”. However, it is only after we initiate the second-level GLM analysis that FSL warps the cope and varcope files using transform matrices generated from the first-level, creating a subfolder that is used for the fixed-effect modeling. Since the fixed-effect model averages across two runs, our major outputs at this level are likewise four copes and four varcopes for each participant. Again, a log file is generated by FSL at this stage.

**Figure 2:**
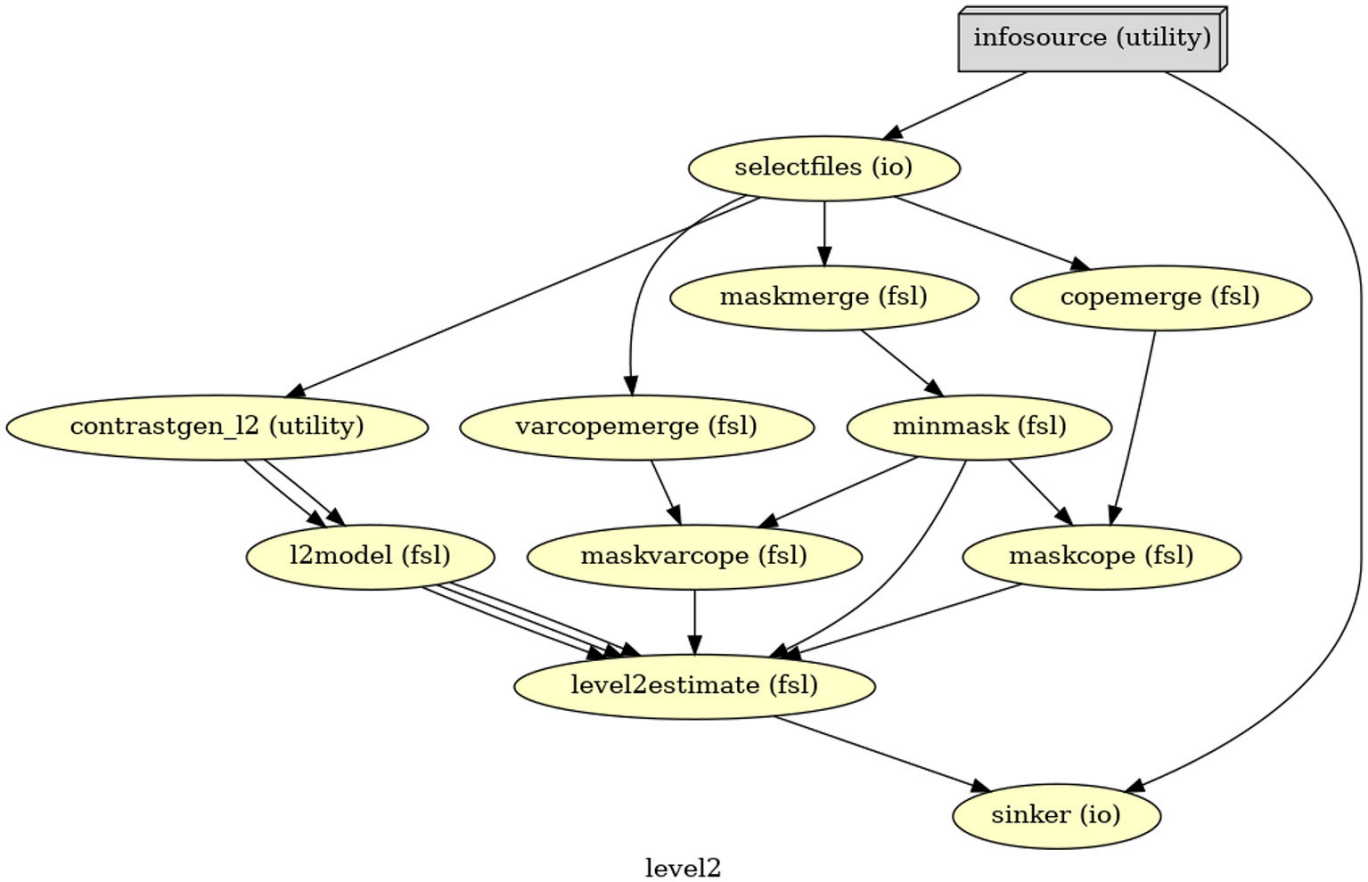
The directed acyclic graph (DAG) for the second-level GLM in Nipype.

In Nipype, similarly, we set up the warping at the end of the first-level GLM and import the warped copes and varcopes into the second level GLM. The second-level GLM in Nipype (Figure 2) then includes merging two copes and two varcopes, respectively, masking the merged copes and varcopes, and fitting the fixed-effect model. We only keep the relevant outputs (one cope, one varcope, and one mask for each participant) for the third-level GLM analysis.

### The third-level GLM

The third-level (cross-subject) GLM is also known as a group-level analysis and has two central steps: (1) choosing a statistical model based on the experimental design, and (2) choosing a thresholding method (including multiple comparison correction) for determining the statistical significance of the results. A mixed-effect model is normally used in the group analysis to make results generalizable to the population. Given that the experiment here is a single-group design, one sample *t*-test^12^ was used for group inference. For thresholding, we kept the default setting—cluster-level correction at *Z* = 3.1 (*p* < 0.005). The inputs of the third-level GLM are the results from the second-level GLMs.

In the FSL GUI, we specified the number of inputs (participants) as 26, specified the input data path, set up the output directory, selected “Mixed effects: FLAME 1”, and used “single group average” in the “Model Setup Wizard”. For everything else, we chose default settings. FSL completes the third-level GLM with results written in one top folder encompassing four subfolders, one for each of the four contrasts defined at the first level. Each subfolder contains parameter estimates (e.g., cope and varcope) as well as cluster level corrected results. The corresponding log file can be found in the top folder.

**Figure 3:**
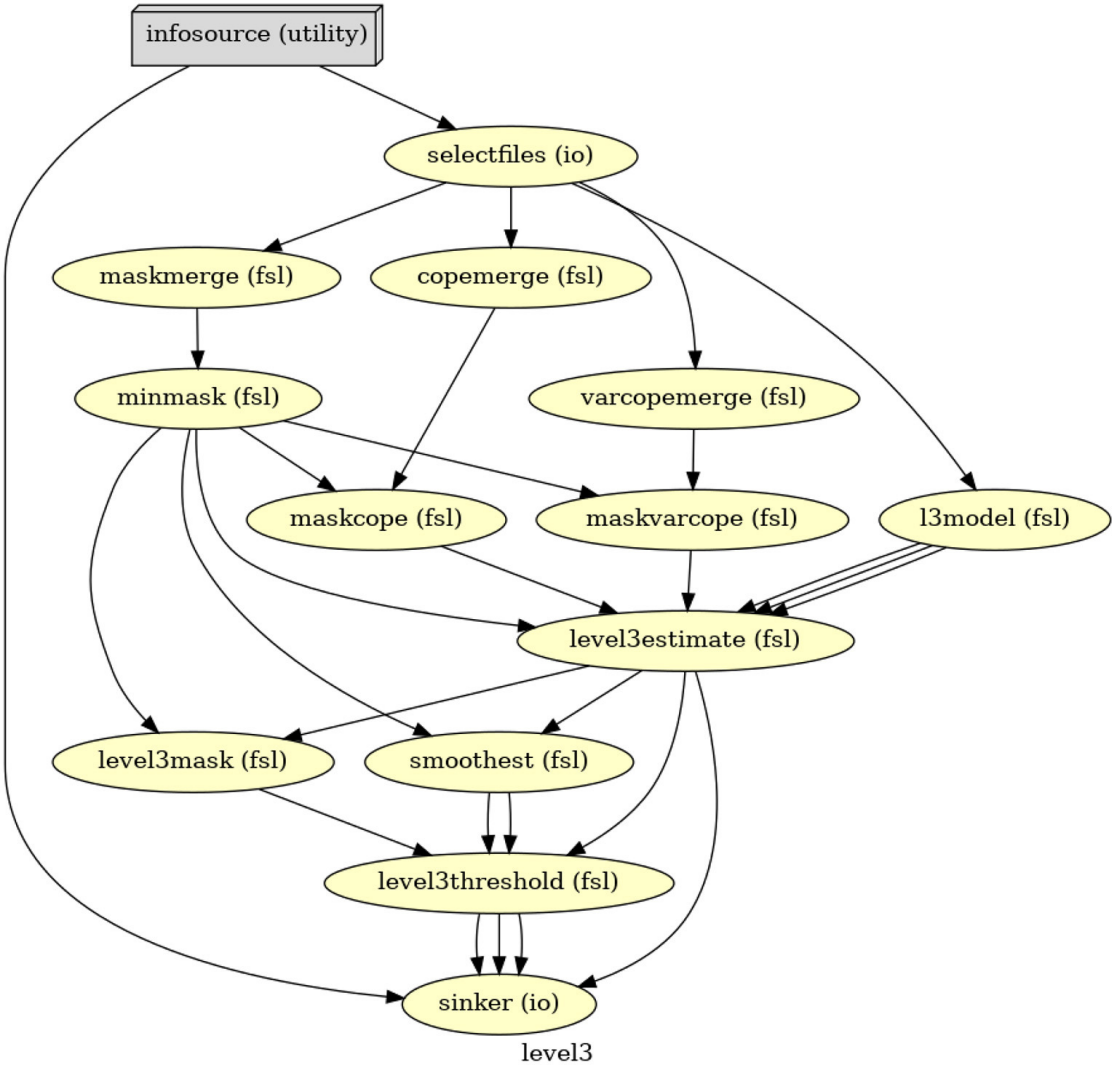
The directed acyclic graph (DAG) for the third-level GLM in Nipype.

The third-level GLM in Nipype starts with merging 26 copes and 26 varcopes for the single group averaging, then specifying the model and fitting it, and lastly correcting the results at the cluster level (Figure 3). The output contains both raw and corrected parameter estimates for each of the four contrasts specified on the first level.

### Output comparison

We conducted detailed comparisons for all outputs from the FSL GUI and from Nipype. For the first-level GLM, we did stepwise comparisons as there was data preprocessing involved and even a slight difference in output for a few voxels at one of the preprocessing steps caused major result deviations for subsequent preprocessing steps. Our stepwise comparisons converted imaging files into arrays and used the Python package *NumPy* to compare (1) number of voxels and (2) value within each voxel^13^. Since FSL rewrites files generated from preceding preprocessing steps, we used shell scripting to create separate files at each preprocessing step in FSL and compared these files with the results from Nipype at each preprocessing step. For the second- and third-level GLMs, we only compared the final output files with the results. The shell scripts, the comparison notebook, and all files we used for the comparisons are available in our OSF repository.

## Results and troubleshooting

Overall, our three-level GLM pipeline in Nipype provided here successfully reproduced the results in FSL, but only after extensive troubleshooting and significant adjustments that are not documented in available Nipype tutorials. In this section, we visualize results from our Nipype pipeline and the GLMs we ran *via* the FSL GUI that turned out to be identical. Next, we point out the difficulties that we have encountered in producing identical results. We conclude the section with describing the adjustments that were necessary to obtain identical results. In addition, we offer our three-level GLM pipeline in Nipype as a sharable and re-executable notebook and as a solution to the problem of deviating results when using the FSL *via* its GUI or *via* Nipype.

### The first-level GLM

The first-level GLM was conducted on each of two runs for all 26 participants and contained four contrasts (incongruent > baseline, congruent > baseline, incongruent > congruent, and congruent > incongruent). For each contrast, our Nipype pipeline produced the same cope and varcope files (i.e., identical values at the voxel-level) as the first-level GLM *via* the FSL GUI. Similar to the first-level stepwise comparison, we converted the statistical map files into arrays and used *NumPy* to compare (1) number of voxels and (2) value within each voxel.

There were two major obstacles that initially impeded the reproduction of the first-level GLMs in Nipype. First, FSL performs more calculations in its backend than it explicitly presents in its GUI. For example, the first step in the first-level GLM—*initialization* (i.e., converting functional images to float representation and extracting the middle volume of the first run as the reference)—is not documented in the GUI; one can only find this step in the log file. Similarly, most settings in the FSL GUI lead to multiple undisclosed computations, which are not automatically reproduced when using FSL *via* Nipype. Without a full understanding of FSL's computing logic, a successful reproduction *via* Nipype is impossible to achieve. In this regard, we made use of FSL's log files and the FSL UserGuide^14^ to precisely reconstruct every analytic step of FSL's first-level GLMs in Nipype. Nevertheless, we ran into additional troubles with our goal to obtain identical results in Nipype and FSL. In the FSL GUI where SUSAN noise reduction is specified, a brightness threshold (*bt*) is needed and estimated from a FWHM smoothing kernel. Surprisingly, even a slight difference in *bt* can lead to very different results in the final stage of the GLM analysis. We used FWHM as 5 mm in FSL and obtained a *bt* of 2.12314225053. Yet, Nipype uses a different algorithm [*bt* = *float (fwhm)/np.sqrt (8* ^*^ *np.log (2))*] to calculate *bt* from FSL. Therefore, to acquire the same *bt*, FWHM should be set as 4.9996179300001655 in Nipype rather than 5. This issue could not be identified until we did a stepwise outcome comparison.

**Figure 4:**
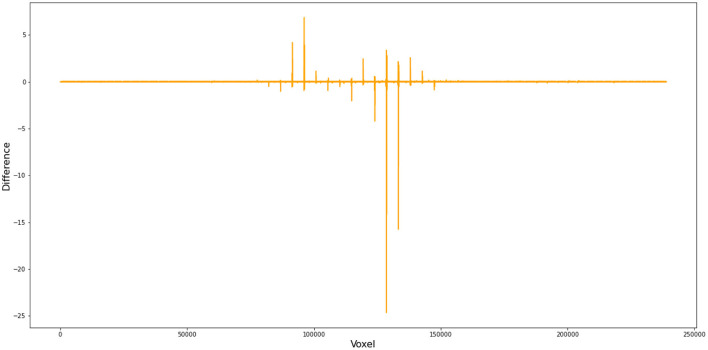
Voxel-wise difference between FSL and Nipype cope results. In this comparison, we used FWHM = 5 mm instead of 4.9996179300001655 in Nipype during preprocess and obtained cope files of the first level GLM. This figure demonstrates the difference in the cope file of the first contrast (incongruent > baseline) between FSL and Nipype outputs for subject 11, run 1.

To better demonstrate how this small difference in FWHM can cause substantial deviations in the first-level GLM results, we compared (1) the files from FSL and Nipype at the step of SUSAN noise reduction and (2) the cope files from FSL and Nipype when setting FWHM as 5 instead of 4.9996179300001655 in Nipype. Our numerical comparison shows that (1) there are 27,981,621 out of 34,887,430 voxels that have different values after SUSAN noise reduction^15^ and (2) there are 223,069 out of 238,955 voxels different in the cope file at the first level^16^ (Figure 4).

### The second-level GLM

The second-level GLM used a fixed-effect model to average parameter estimations across two runs within each subject. Our Nipype pipeline managed to produce the same results (i.e., identical value at the voxel level) as FSL GUI after a few adjustments.

There were two problems that emerged with respect to the registration step and the general linear model setup. Before any processing happens at the second level, FSL first transforms the results (e.g., copes and varcopes) from the first-level into a standard space. Again, this step happens in FSL's backend and has no representation in FSL's GUI. In the FSL log file, this registration procedure is performed through the function *featregapply*. However, there is no *featregapply* in Nipype; instead a function called *FEATRegister*^17^ is used, which can register FEAT directories to a specific standard. The problem is that the outputs of the first-level GLM in Nipype are not structured as FEAT directories. To solve this registration problem, we chose an alternative approach. Instead of setting up the second-level GLM with all 26 participants, we just used two participants^18^. The resulting FSL log file then provided the details under *featregapply* that *FLIRT* indeed used for transformation for all participants. We then adopted this function in Nipype, applied it to all 26 participants, and eventually obtained the same standard images as the FSL GUI does.

In setting the fixed-effect model, FSL averages the first-level results from two runs for each participant, and this procedure is conducted for all participants simultaneously. However, Nipype recommends in its documentation using the *L2Model*^19^ to set up the model, which generates subject-specific second-level estimates. In other words, L2Model performs the second-level analysis on each participant separately. Instead of adopting L2Model, we chose to maintain consistency with the FSL GUI bye using *MultipleRegressDesign*^20^, which, as its name indicates, can simultaneously perform the estimation on all 26 participants.

### The third-level GLM

After the adjustments on the first- and second-level outlined above, no further adjustments had to be made in Nipype for the third-level GLM analysis; we obtained identical copes in Nipype and *via* the FSL GUI before and after thresholding.

## Lessons learned and discussion

Our work offers a well-documented pipeline and environment for reproducing a full three-level GLM analysis *via* the FSL GUI in Nipype. As one of the most widely used statistical models in fMRI analysis, GLM analyses still play an important role in studying human cognition. Typically, GLMs in FSL are mainly executed through its GUI, which may impede reproducibility as analysts can fail to precisely document the options they have selected. Even if analysts document their selected settings, these settings can lead to different computational outputs that do not always correspond to what Nipype uses by default in its FSL interface nodes. One possible solution could be to always provide FSL log files in addition to the results produced by the FSL GUI or use FSL command line scripts instead. However, as we have shown, FSL commands in its log file cannot always be easily translated back to GUI options and vice versa. Our adjusted Nipype pipeline for a complete three-level GLM analysis provides an alternative to both the “black box” nature of a GUI-based approach and the less accessible nature of a command-line script. Our step-by-step pipeline clearly demonstrates how the input data are arranged, transformed, calculated, and grouped into the outputs. Moreover, Python, which relies on English-like syntax, makes those logical transitions within the computing procedure easier to grasp. Therefore, our pipeline not only contributes to increasing reproducibility in neuroimaging studies, but also serves as an educational and instructive tool for a better understanding of brain imaging analyses.

Importantly, however, given the obstacles we have encountered when reproducing results from FSL's GUI in Nipype, we recommend using Nipype with some caution and always providing complete documentation of code and procedures to promote reproducibility. We also recommend using our adjusted Nipype pipeline for three-level GLM analyses. With the original Nipype paper (Gorgolewski et al., 2011) as the seed paper, we selected the top 5% (*N* = 51) cited publications that cited the seed paper or used Nipype for their data analyses and recorded whether analytical pipelines were provided. We found that only 35.3% (*N* = 18) of those 51 articles made their code available. Fellow researchers who have used Nipype pipelines for GLM-based analyses with standard setups using Nipype's available documentation may want to check their results and evaluate to what extent the results deviate from the results obtained by the FSL GUI. Of course, this suggests that the FSL GUI is the standard for result accuracy and valid result interpretations, which can be likewise questionable. In any case, as surely a substantial number of analysts still use the FSL's GUI on a regular basis for their analyses and result interpretations, Nipype analysts who strongly support reproducibility of results across analytical platforms may want to produce workflows that match the results of the corresponding analytical interfaces when used separately from Nipype.

## Data availability statement

The code and supplementary material which supports the findings of this study are available via OSF (https://osf.io/prg53/) and Github (https://github.com/medianeuroscience/nipype_repro).

## Ethics statement

Ethical review and approval was not required for the study on human participants in accordance with the local legislation and institutional requirements. Written informed consent from the patients/participants legal guardian/next of kin was not required to participate in this study in accordance with the national legislation and the institutional requirements.

## Author contributions

YC and RW conceived the idea. YC developed the first draft and ran the initial analyses. All authors contributed equally to revising the initial draft and adding to the analyses.

## Funding

Funding for this study was received by UCSB's Open Access Publishing Fund.

## Conflict of interest

The authors declare that the research was conducted in the absence of any commercial or financial relationships that could be construed as a potential conflict of interest.

## Publisher's note

All claims expressed in this article are solely those of the authors and do not necessarily represent those of their affiliated organizations, or those of the publisher, the editors and the reviewers. Any product that may be evaluated in this article, or claim that may be made by its manufacturer, is not guaranteed or endorsed by the publisher.

## Footnotes

^1^ https://miykael.github.io/nipype_tutorial/

^2^ https://dpaniukov.github.io/

^3^ https://github.com/poldracklab/ds003-post-fMRIPrep-analysis

^4^ https://osf.io/prg53/

^5^ https://openneuro.org/datasets/ds000102/versions/58016286cce88d0009a335df

^6^ https://andysbrainbook.readthedocs.io/en/latest/fMRI_Short_Course/fMRI_Intro.html

^7^ https://github.com/ReproNim/neurodocker

^8^More details see our OSF repository, https://osf.io/prg53/?view_only=9d7974834a484cdb972bcc3989589b78.

^9^More details see https://andysbrainbook.readthedocs.io/en/latest/fMRI_Short_Course/fMRI_06_Scripting.html.

^10^More details see https://fsl.fmrib.ox.ac.uk/fsl/fslwiki/Atlases.

^11^ https://fsl.fmrib.ox.ac.uk/fsl/fslwiki/FEAT/UserGuide#FEAT_Output

^12^ https://fsl.fmrib.ox.ac.uk/fsl/fslwiki/FEAT/UserGuide#Single-Group_Average_.28One-Sample_T-Test.29

^13^Code can be found in *nipype fsl comp.ipynb* fron our OSF repository.

^14^ https://fsl.fmrib.ox.ac.uk/fsl/fslwiki/FSL

^15^See Step 11 in *nipype fsl comp.ipynb* fron our OSF repository.

^16^See Step 15 in *nipype fsl comp.ipynb* fron our OSF repository.

^17^ https://nipype.readthedocs.io/en/latest/api/generated/nipype.interfaces.fsl.model.html#featregister

^18^The minimal number of input files in FSL is two, and one participant only have two copes for each contrast, which is not enough, so we set the model up with two participants.

^19^ https://nipype.readthedocs.io/en/latest/api/generated/nipype.interfaces.fsl.model.html#l2model

^20^ https://nipype.readthedocs.io/en/latest/api/generated/nipype.interfaces.fsl.model.html#multipleregressdesign
